# Around the Hospitals

**Published:** 1897-07-03

**Authors:** 


### AROUND THE HOSPITALS.

**Leeds General Infirmary.**—Taking the financial aspect of the year 1896 first, we find that, compared with 1895, the receipts show a decrease of £314, the figures being £23,442 and £23,756 respectively. In 1895, however, £1,300 was received from the Leeds Musical Festival, and if this is not taken into account the 1896 receipts are £986 in excess of those of 1895, an increase which is almost entirely due to the large increase of some £700 in the sum contributed by the workpeople of Leeds (£5,200). The ordinary expenditure was £23,093, against £22,475 the previous year, an increase of £618, mainly accounted for by increases under the head of provisions, stimulants, drugs, and salaries. By a curious coincidence the number of new in-patients admitted during 1896 (5,781) was the same as the number admitted in 1895. To these must be added the 335 cases in the infirmary on the first day of the year, making a total of 6,116 patients treated in the wards during the year. The daily average number of beds occupied was 358 (against 360 in 1895); 17 patients on an average occupied each bed in the year; the stay of a patient in the hospital averaged three weeks. The death-rate amongst the patients was 6·44 per cent., but deducting the large number of patients (112) who died within 48 hours after admission the percentage is reduced to 4·69. The total number of out-patients treated (including 156 in the electrical department and 475 maternity cases) was 43,701, or an average weekly attendance of 2,742. In other words, excluding Sundays, 456 persons attended the out-patient department each day. Adding the 358 patients who, on an average, occupied the beds in the hospital, we arrive at this—that on each week-day in the year the hospital treated over 800 persons for ailments more or less serious. An interesting point is the fact that whilst 50 per cent. of the admissions to the wards were men, only 3 per cent. of the out-patients are placed in that category, the remaining 67 per cent. being women and children. The average cost of each in-patient for treatment, nursing, and maintenance is worked out by the hospital authorities at £2 11s. 10½d., or 17s. 3½d. a-week, while the cost of treating an out-patient is estimated at 1s. 6d. As showing the increased work which has been thrown on this infirmary the following figures are of interest: In 1866 the infirmary spent £5,712 on the relief of 6,837 patients (1,661 in and 5,176 out); ten years later, *i.e.*, in 1876, the expense had increased to £17,085 and 13,985 patients (2,876 in and 11,109 out) were treated; in 1886 the expense reached £15,483 and 25,354 patients (4,167 in and 21,187 out-patients) were under treatment; whilst in 1896 £24,921 was expended on the treatment of 43,863 patients (*i.e.*, 5,781 in and 38,082 out). Two points will be noticed in these figures—first, that the expense in 1876 is much larger than that for 1886, but this is an accidental circumstance, as the average expenditure at that time was about £14,000; and, secondly, that the figures we give for 1896 do not coincide with those given at the commencement of this article. This is so because in the expenditure is included that of the Ida Hospital, and the patients include only the new cases treated, those remaining from the previous year being excluded. This infirmary is one of the few hospitals in Great Britain which boasts the possession of a horse ambulance. It has been much in request during the year, having been called out on 580 occasions, or 90 more than in 1895. We cannot close this *resumé* of the work done by the hospital in 1896 without giving a word of praise to the report. It is one of the most complete reports we have the pleasure of handling, and may well be used as a model by many other large hospitals in the United Kingdom. It has one defect, however, and that is—the accounts are not made upon the Uniform system. The adoption of this form of accounts would not entail much difficulty, as, so far as we can judge, most of the details are already given in the form used by the hospital, and the only labour involved would, therefore, be a rearrangement of the items on the Uniform system.

